# Microglial/Macrophage polarization and function in brain injury and repair after stroke

**DOI:** 10.1111/cns.13620

**Published:** 2021-03-01

**Authors:** Junxuan Lyu, Di Xie, Tarun N. Bhatia, Rehana K. Leak, Xiaoming Hu, Xiaoyan Jiang

**Affiliations:** ^1^ Department of Neurology Pittsburgh Institute of Brain Disorders & Recovery University of Pittsburgh Pittsburgh PA USA; ^2^ Graduate School of Pharmaceutical Sciences School of Pharmacy Duquesne University Pittsburgh PA USA; ^3^ Geriatric Research Education and Clinical Center Veterans Affairs Pittsburgh Health Care System Pittsburgh PA USA

**Keywords:** neuroinflammation, phagocytosis, polarization, repopulation, transplantation

## Abstract

Stroke is a leading cause of disability and mortality, with limited treatment options. After stroke injury, microglia and CNS‐resident macrophages are rapidly activated and regulate neuropathological processes to steer the course of functional recovery. To accelerate this recovery, microglia can engulf dying cells and clear irreparably‐damaged tissues, thereby creating a microenvironment that is more suitable for the formation of new neural circuitry. In addition, monocyte‐derived macrophages cross the compromised blood‐brain barrier to infiltrate the injured brain. The specific functions of myeloid lineage cells in brain injury and repair are diverse and dependent on phenotypic polarization statuses. However, it remains to be determined to what degree the CNS‐invading macrophages occupy different functional niches from CNS‐resident microglia. In this review, we describe the physiological characteristics and functions of microglia in the developing and adult brain. We also review (a) the activation and phenotypic polarization of microglia and macrophages after stroke, (b) molecular mechanisms that control polarization status, and (c) the contribution of microglia to brain pathology versus repair. Finally, we summarize current breakthroughs in therapeutic strategies that calibrate microglia/macrophage responses after stroke.

## Abstract 2

The present review summarizes recent advances in microglial research in relation to stroke with emphases on microglial/macrophage phenotypic polarization and function in brain injury and repair. It also reviews the physiological characteristics and functions of microglia in the developing and adult brain, and describes current breakthroughs in therapeutic strategies that calibrate microglia/macrophage responses after stroke.

## Graphical abstract

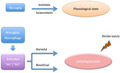

## 1 INTRODUCTION

Stroke is a leading cause of long‐term disability and mortality across the globe. There are two major types of stroke: ischemic stroke and intracerebral hemorrhage (ICH). Ischemic stroke accounts for ~70%–80% of all stroke cases, and ICH accounts for ~10%–20%.^1^ Ischemic stroke and ICH trigger blood‐brain barrier (BBB) disruption, neuroinflammation cascades, and neuronal death, leading to severe neurological deficits.^2^, ^3^ Treatments against both the acute and chronic phases of stroke are limited,^4^, ^5^, ^6^ necessitating research into therapeutic strategies to attenuate brain injury and facilitate functional recovery in stroke patients. The cellular and molecular mechanisms that determine stroke onset and injury progression are only beginning to be identified with the help of modern genetic research tools.

Microglia, the resident immune cells of the CNS, are widely distributed across the brain and spinal cord and form an integral part of the neurovascular unit. One of the major functions of microglia is to constantly survey the CNS microenvironment and maintain brain homeostasis.^7^, ^8^ Stroke injury induces rapid activation and migration of microglia to the lesion sites.^9^ Microglia can undergo profound morphological changes (from ameboid to hyper‐ramified) and phenotypic polarization (from quiescent to active), culminating in the release of cytokines, chemokines, and other immune modulators. Microglial functions are largely dependent on their pro‐ or anti‐inflammatory phenotype at specific pathophysiological stages and in specific brain regions after stroke, underscoring the importance of both spatial and temporal dimensions in microglia dynamics.^7^ In general, pro‐inflammatory microglia secrete detrimental cytokines and molecules that aggravate brain injury, whereas anti‐inflammatory microglia promote brain repair and facilitate neurological recovery.^10^ As microglia harbor the inherent mechanisms to rapidly switch between detrimental *vs*. beneficial functions, modulating these characteristics may be suitable for the development of novel therapeutics for stroke. In this review, we summarize microglial biology and discuss their polarization and modulatory mechanisms, both in terms of brain injury during stroke and brain restoration during the post‐stroke recovery phase. Lastly, we describe recent breakthroughs in therapeutic strategies that target microglial responses after experimental stroke.

## 2 PHYSIOLOGICAL CHARACTERISTICS AND FUNCTIONS OF MICROGLIA IN THE BRAIN

### 2.1 Morphology of microglia in the developing and adult healthy brain

Microglia were first defined by Pío Del Río‐Hortega over a century ago.^11^ Microglial cells first develop from primitive myeloid progenitors in the yolk sac and migrate to the CNS from embryonic day 8.5, until BBB closure on embryonic day 13. In the embryonic and early developmental stages, microglial development and differentiation precedes other CNS cells.^12^ Microglia harbor a unique ameboid morphology during fetal brain development, and have distinct molecular and functional properties compared to adult microglia.^13^

In the healthy adult brain, microglia numbers and spatial distribution are maintained by a process of self‐renewal.^14^ The density of microglia is region‐specific, with higher numbers in the hippocampus and olfactory bulb, and lower numbers within fiber tracts.^15^ There is also substantial variability in the size and morphology of microglia in different brain regions.^16^ For example, microglia in gray matter have smaller somas than white matter, with process extensions. Microglia continually extend and retract these branches during CNS surveillance.^17^ To facilitate migration from gray matter into white matter tracts, microglia transform from a ramified into an amoeboid shape, with rounded cell bodies and processes that orient along the direction of the fibers.^18^, ^19^ After exposure to challenges in the microenvironment, microglia transform into a more spherical shape and spring into action against toxic stimuli.^17^, ^20^ Aside from exposure to environmental challenges, aging also impacts microglial morphologies, with natural shortening of processes and an increase in somal volumes in aged brains.^21^

### 2.2 Microglial function in the developing and adult healthy brain

Microglia maintain CNS homeostasis in both adult and developing brains. Microglia are aptly named the “engineers” of the CNS, as they participate in neurogenesis, programmed cell death, synapse elimination, and neural circuit formation during development.^22^, ^23^ During early development, neurotrophic factors secreted by microglia enhance neurogenesis and promote the survival and differentiation of specific neuronal lineages.^24^, ^25^ Additionally, immature neurons that undergo programmed cell death are cleared by microglia without triggering inflammatory processes.^26^, ^27^ Microglia also remodel the CNS by selectively pruning redundant neuronal processes and defective synapses that have the potential to hinder the maturation of neuronal circuitry.^28^, ^29^ Ultimately, the physiological roles of microglia help establish and strengthen functional and mature neuronal circuits during brain development.

In adult brains, microglia actively scan the brain microenvironment by extending and retracting their processes. Alterations in the brain microenvironment elicit rapid and profound changes in microglial morphologies and functions, inducing the expression of several pro‐survival molecules. Microglia also actively interact with neurons, influence neuronal regeneration, proliferation, migration, and secrete neurotrophic factors, such as insulin‐like growth factor‐1 (IGF‐1), to promote the health and survival of neighboring neurons.^30^

Under pathological conditions, microglia are a critical component of the innate immune sentinel network and serve as the first line of defense in the CNS. Phagocytosis is an established microglial function designed for removal of cellular debris or dead cells, without necessarily initiating an inflammatory response.^31^, ^32^ Microglia quickly detect pathogens, including bacteria, fungi, and viruses that enter the brain parenchyma and respond by producing pro‐ and/or anti‐inflammatory cytokines, chemokines, and complement proteins. These reactions are designed to promote pathogen clearance, although at times may cause intense, collateral inflammatory reactions and exacerbate CNS pathology. On the other hand, the collateral inflammatory reactions and highly reactive cellular milieu may be essential to block and clear pathogenic infections and prevent organismal death.

## 3 MICROGLIAL POLARIZATION AND THEIR MODULATORY MECHANISMS AFTER STROKE

### 3.1 Microglial activation and phenotypic polarization after stroke

Microglia are among the first cells to respond to acute brain injury, with their activation lasting months after injury onset. In a transient ischemic stroke model, activated Iba1^+^ microglia emerge in the infarct core area within 24 hours and peak within 4–7 days after reperfusion. In the peri‐infarct region, microglia accumulate much earlier, within ~3.5 hours and peak at 7 days post‐reperfusion.^33^ Activated microglia undergo rapid morphological changes, including thickening of processes and hypertrophy of somata. Activated microglia also upregulate cell surface markers and secrete pro‐ or anti‐inflammatory cytokines. Early studies classified activated microglia into “detrimental” M1 versus “protective” M2 subtypes according to their protein/cytokine expression profiles. M1 microglia secrete pro‐inflammatory cytokines, such as TNF‐α, IL‐1β, IL‐12, and IL‐23, and they can be detected by cell surface markers such as CD16, CD32, and CD86. In contrast, M2 microglia secrete anti‐inflammatory cytokines such as TGF‐β, IL‐4, IL‐10, IL‐13, and growth factors such as vascular endothelial growth factor (VEGF), brain‐derived neurotrophic factor (BDNF). In general, M2 microglia can be identified by CD206 and Arg1, among other markers.^34^, ^35^ The view that microglia are dichotomous and exclusively express *either* M1 or M2‐specific markers is the subject of recent controversy and regarded as oversimplified. For example, in the case of traumatic brain injury, M1/M2 microglial polarization may be concurrent^36^ and with the emergence of single‐cell technologies and mass cytometry, novel microglial subtypes with distinct and complex molecular signatures have been identified.^37^, ^38^ Despite these observations and critiques, the terms “M1” and “M2” continue to be useful in distinguishing microglial phenotypes based on protein marker expression or pro‐ versus anti‐inflammatory functional effects.

**FIGURE 1:**
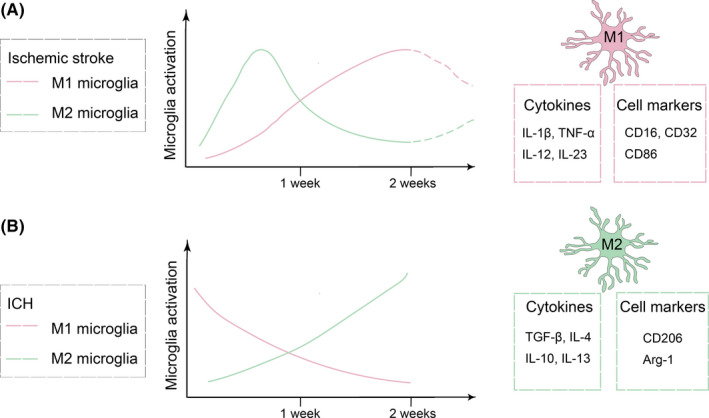
Temporal activation and polarization of microglia in ischemic stroke and intracerebral hemorrhage. (A) In ischemic stroke, microglia gradually switch from M2‐phenotype toward M1‐phenotype within 1 week after injury. (B) In the intracerebral hemorrhage (ICH) model, microglia with M1‐phenotype exhibit a decreasing trend, while M2‐like microglia exhibit an increasing trend in the first two weeks after injury. A few cell markers and cytokines expressed by M1 and M2 microglia are also shown

Several groups have examined the spatiotemporal activation and polarization of microglia after stroke.^39^, ^40^ In ischemic stroke, activated microglia express M2 phenotypic markers in the acute stage. However, they gradually switch toward M1 phenotype at about 1 week and last for a few weeks after injury (Figure 1).^41^ This phenotype shift may result from the recruitment of M1 microglia/macrophages to the injury site and the M2‐to‐M1 transition of local activated microglia/macrophages. In ICH, an M1 to M2 microglial/macrophage transition is observed in both collagenase‐induced ICH and blood‐induced ICH.^42^ This transition usually happened within 1 week after injury and can last for at least 14 days after injury (Figure 1).^42^ The reasons for the transition need further studies, but one of the major possibility might be the infiltration of M2‐like circulating blood monocytes or macrophages.

Imaging techniques such as magnetic resonance imaging (MRI) and positron emission tomography (PET) have enabled the examination of microglial activation in clinical stroke. Although patient brains showed activated microglia in acute, sub‐acute, and chronic phases after ischemic stroke,^43^ the specific activation patterns in the infarct core and peri‐infarct regions vary across individuals. In some patients, microglia are initially chiefly activated in the ischemic core within 24 to 48 hours after stroke and only then gradually extend to the peri‐infarct area.^44^ In another study, activated microglia increased in both the infarct core and peri‐infarct areas 7 days after stroke and the activation gradually decreased over time.^45^ These divergent patterns of microglial activation may reflect differences in the initial severity of the injury, the status of the BBB in the ischemic core versus penumbra, and the absence or presence of sepsis in stroke patients. This clinical research area warrants further exploration. For example, clinician scientists might examine microglial activation in stroke patients early after injury and correlate these spatiotemporal patterns with subsequent neurological outcomes.

### 3.2 Molecules and mechanisms modulating microglial phenotype polarization after stroke

**FIGURE 2:**
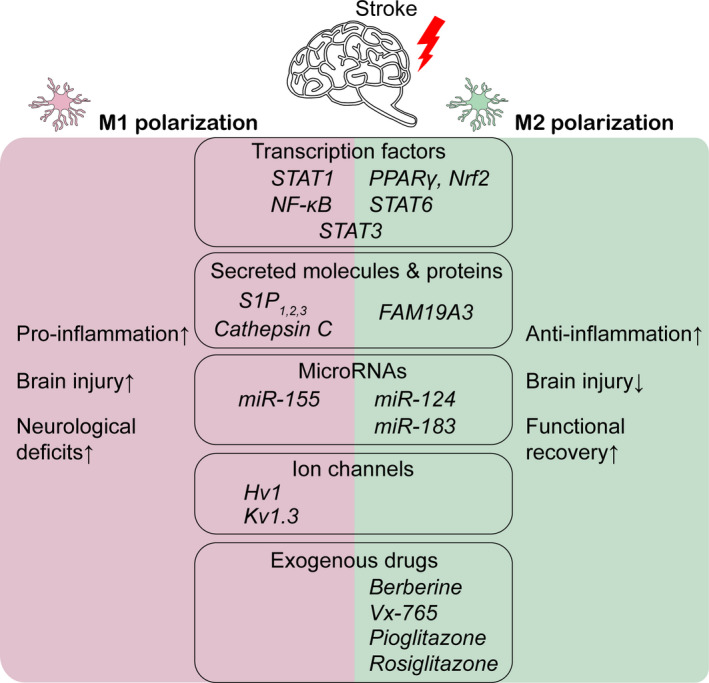
Modulators of microglia phenotype polarization after stroke. Molecules stimulating microglia toward M1 pro‐inflammatory polarization may exacerbate brain injury and increase neurological deficits. Anti‐inflammatory M2 microglia attenuate brain injury and promote functional recovery after stroke. This list is not meant to be exhaustive

Below, we have summarized some of the more widely reported modulators of microglial phenotypic polarization post‐stroke injury (Figure 2).

#### 3.2.1 Transcription factors

Peroxisome proliferator‐activated receptor gamma (PPARγ) belongs to a superfamily of nuclear receptors that contribute to antioxidant and anti‐inflammatory responses.^46^ PPARγ agonists such as rosiglitazone^47^ or PPARγ activating factors, such as 1, 25‐dihydroxyvitamin D3 (1, 25‐D3), recombinant human fibroblast growth factor 21 (rhFGF21), and oleic acid^48^, ^49^, ^50^ attenuate brain inflammation and protect against ischemic injury in murine models of stroke. Specifically, microglia respond with a decrease in M1 phenotypic markers and a parallel increase in M2 markers after PPARγ activation. In ICH induced by intraparenchymal injections of blood, treatment with PPARγ agonists decreased M1 cytokine levels and promoted microglial phagocytosis of extravasated red blood cells, thereby mitigating the cytotoxic insult and encouraging hematoma resolution.^51^, ^52^

Members of the signal transducer and activator of transcription (STAT) family of transcription factors play key roles in cellular proliferation, immunity, and inflammation.^53^, ^54^ STAT1 controls M1 microglia/macrophage activation,^55^ whereas STAT6 promotes M2‐phenotypic polarization^56^ after cerebral ischemia. The effect of STAT3 is likely to be context‐dependent: In mice with collagenase‐induced ICH, phosphorylated (active) STAT3 was mainly present within activated microglia and macrophages, and inhibition of pSTAT3 reduced brain edema.^57^ Conversely, microglia may be propelled toward the protective, anti‐inflammatory M2‐phenotype by modulating the STAT3 signaling pathway in ischemic injury models.^58^, ^59^

Other transcription factors implicated in microglial polarization after stroke injury include the NF‐κB and Nrf2‐related signaling pathways. Nuclear factor‐κB (NF‐κB) is a transcription factor that regulates the expression of M1‐signature genes. Activation of NF‐κB by toll‐like receptors (TLR2 and TLR4) increases pro‐inflammatory cytokine levels in ischemic stroke and ICH.^60^, ^61^, ^62^, ^63^ The nuclear factor erythroid 2‐related factor 2 (Nrf2) and its signaling pathway encourages microglia/macrophage polarization toward the M2 phenotype in ischemic stroke and ICH.^64^, ^65^ Hence, the net balance between NF‐κB versus Nrf2 pathway activation may partly determine the course of the injury and recovery phases after stroke.

#### 3.2.2 MicroRNAs

MicroRNAs (miRNAs) are small, non‐coding RNAs that bind to mRNAs and play important roles in the regulation of gene expression at the post‐transcriptional level. A number of studies have elucidated the role of specific miRNAs in microglia polarization.^66^, ^67^, ^68^ For example, miR‐124 modulates and shifts microglia from M1 to M2 phenotype in the sub‐acute phase after stroke^66^ miR‐155, which is a well‐established microglia phenotype modulator, could promote M1 polarization after stroke.^69^ miR‐183 reduces pro‐inflammatory IL‐1β, IL‐6, and TNF‐α in microglia via NF‐κB signaling in the middle cerebral artery occlusion (MCAO) model.^67^

#### 3.2.3 Secreted molecules and proteins

Sphingosine 1‐phosphate (S1P) is a lipid mediator secreted into the extracellular environment to regulate cell migration, differentiation, and survival^70^ and has emerged as a microglia modulatory target in stroke.^71^, ^72^, ^73^ In a brain ischemia model, signaling through the S1P_1_ receptor triggers microglial activation and impacts microglial polarization.^71^ An antagonist of S1P_1_ activity, AUY954, attenuated the activation of M1‐relevant molecules ERK1/2 and p38, but increased activation of the M2‐relevant, Akt molecule.^71^ Similarly, the S1P_2_‐specific receptor antagonist JTE013 suppressed M1‐relevant NF‐κB, ERK1/2, and JNK activation in activated microglia.^72^ The S1P_3_ inhibitor CAY10444 mitigates ischemia‐induced neurological deficits, reduces microglia activation, and impacts pro‐inflammatory M1 polarization as well as phosphorylation of ERK1/2, p38 MAPK, and Akt.^73^ Other secreted molecules also regulate microglia phenotype polarization. FAM19A3, a secreted protein in the brain, promotes polarization toward the M2 phenotype and ameliorates cerebral ischemia.^74^

#### 3.2.4 Ion channels

The ion channels Hv1 and Kv1.3 also regulate microglia polarization and inflammation.^75^, ^76^ Hv1^−/−^ mice exhibit smaller infarcts and superior neurological performance, which are associated with a microglial polarity switch from M1 to M2.^75^ Genetic knockout of Kv1.3 in microglia reduces the expression of LPS‐induced pro‐inflammatory mediators, including IL‐1β, TNF‐α, IL‐6, and iNOS, and reduces the LPS‐induced impairment in hippocampal long‐term potentiation.^76^

#### 3.2.5 Exogenous drugs

Several exogenous compounds and drugs are able to modulate microglial phenotype in experimental studies.^77^, ^78^, ^79^, ^80^ For example, the naturally occurring chemical compound berberine may protect against ischemic stroke by modulating microglial polarization.^77^, ^78^ Vx‐765, a small‐molecule caspase‐1 inhibitor, mediates M2 microglia/macrophage polarization by suppressing NF‐κB activation.^79^ As mentioned previously, widely used PPARγ agonists such as pioglitazone and rosiglitazone can propel microglia toward anti‐inflammatory phenotypes.^81^ The repurposing of these drugs may pave a new avenue for stroke treatment.

### 3.3 Contribution of infiltrated peripheral myeloid cells to stroke pathology and repair

Microglia are not the sole myeloid cell in the CNS even under resting, physiological conditions. Although microglia dominate in brain parenchyma, border associated macrophages (BAM) also reside in specialized compartments of the CNS and are named after the locations they inhabit, including leptomeningeal macrophages, choroid‐plexus macrophages, and perivascular macrophages.^82^ CNS myeloid cells are distinct from peripheral myeloid cells in several aspects. Unlike peripheral monocytes that are constantly renewed from bone marrow stem cells, CNS myeloid cells undergo self‐renewal.^83^ In addition, the turnover rate for CNS myeloid cells is much slower than peripheral myeloid cells, rendering them more vulnerable to loss of population numbers from toxic insults. It has also been reported that CD163^+^ BAM are reprogrammed after acute stroke, resulting in leukocyte chemotaxis and neurological impairments.^84^, ^85^ Perhaps for these reasons, BAM depletion reduces acute ischemic brain injury.

Under pathological conditions such as stroke, monocyte‐derived macrophages may cross the compromised BBB and invade the injured brain, thereby diversifying the population of myeloid cells and increasing their functional repertoires. Immature monocytes infiltrate into the infarct border within 24 hours of stroke and differentiate into mature phagocytes within the lesion boundaries.^86^ Peripheral macrophages are most abundant in the ischemic brain within 3–7 days after transient focal cerebral ischemia, and the numbers decrease thereafter.^87^, ^88^ Mice with selective deletion of CCR2, a molecule essential for monocyte infiltration, show a delay, rather than abolishment of post‐stroke inflammation, which is accompanied by reduced angiogenesis and worse neurological performance.^89^ However, further analyses in the same report indicated heterogeneities within the infiltrating CCR2^+^ monocytes, which may contribute to acute neuroinflammation and long‐term functional recovery. A recent study employing Cxcr4‐CreER‐mediated lineage‐tracing mice successfully distinguished hematopoietic stem cell‐derived monocytes from microglia and other tissue‐resident macrophages, thereby identifying critical functions of blood‐borne monocytes in initiation of a robust defense response after acute cerebral ischemia.^90^

Microglia, brain‐resident macrophages, and peripheral monocyte‐derived macrophages occupy distinct functional niches, playing important and diverse roles in the ischemic brain. Distinguishing between these cells has been a technical challenge, as these cells all belong to a large family of mononuclear phagocytes and therefore share commonalities in gene expression. However, with recent advances in single‐cell technologies, several microglia‐specific markers (*eg*, Tmem119, P2ry12, and Sall1) and macrophage‐specific markers (*eg*, Mertk) have been identified and are now used to specify myeloid lineage cells.^91^ Furthermore, a number of new technical approaches can distinguish the roles of microglia and macrophages in stroke. Bone marrow chimeric mice are frequently used to examine the spatial distributions of microglia versus peripheral macrophages in the ischemic brain and to explore the contribution of infiltrating immune cells.^56^, ^92^, ^93^ Recent studies also have leveraged the *cre*‐*loxp* system and the development of novel cell type‐specific *cre* mice to further enhance our ability to distinguish the functions of distinct myeloid cells. For example, Tmem119‐CreER mice and P2ry12‐CreER mice have been generated and are available for brain‐resident microglia‐specific exploration.^94^, ^95^ Cxcr4‐CreER mice are also valuable tools for research on hematopoietic stem cell‐derived monocytes.^90^ In the wake of rapid technological advances, these novel approaches are expected to shed light on mechanistic underpinnings of the different myeloid compartments in stroke injury and post‐stroke brain repair.

## 4 DIVERSE FUNCTIONS OF ACTIVATED MICROGLIA/MACROPHAGES IN STROKE

### 4.1 Impact on BBB integrity

Microglia are vital components of the neurovascular unit and regulate BBB integrity after stroke.^96^ Microglia are activated acutely after stroke and upregulate the expression of transcription factors, including hypoxia‐inducible factor‐1 (HIF‐1) and NF‐κB, as well as reactive oxygen species (ROS) and nitric oxide (NO). The latter molecules contribute to endothelial damage and BBB hyperpermeability.^41^, ^97^ In addition, perivascular microglial/macrophage activation elevates the release of pro‐inflammatory cytokines, such as IL‐6, IL‐1β, and TNFα, all of which are known to be detrimental to BBB integrity during the early phase of ischemia.^98^, ^99^, ^100^, ^101^ Endothelial cells are also activated by these pro‐inflammatory cytokines, leading to an increase in the expression of adhesion molecules, such as intercellular adhesion molecule 1 (ICAM‐1), P‐selectin, and vascular cell adhesion protein (VCAM), which in turn enhance the infiltration of circulating leukocytes into brain parenchyma, leading to further brain inflammation in late phases after stroke.^102^ In addition, *in vivo* two‐photon live imaging suggests that activated microglia/macrophages can migrate to blood vessels and engulf endothelial cells by phagocytosis soon after reperfusion, which causes an eventual breakdown of the BBB.^103^ In contrast, anti‐inflammatory microglia/macrophages may facilitate long‐term neurovascular remodeling and improve neurological functions at late phases after stroke.^104^ A recent study found that depletion of microglia damages BBB integrity after ischemic stroke, perhaps due to the neutrophils recruited into the infarct area, which are not engulfed because of the depletion of microglia.^105^ In sum, these studies reveal diverse roles for microglia in stroke, which may be influenced by injury stage, pro‐ versus anti‐inflammatory status, cell‐cell interactions with non‐microglial cells, and the nature of the surrounding cellular milieu.

### 4.2 Impact on neurogenesis

As mentioned above, microglia density is high in the hippocampus and olfactory bulb, two regions that undergo neurogenesis even into adulthood. Release of pro‐ or anti‐inflammatory cytokines by microglia influences the proliferation and differentiation of neural stem/progenitor cells (NPCs) and neurogenesis after stroke.^106^ In an ex vivo study, live sections from ischemic brains incubated with conditioned media from M2‐microglia showed an increase in the proliferation and differentiation of NPCs within the subventricular zone (SVZ) neurogenic niche.^107^ Strategies to switch microglia/macrophages from the M1 to M2 phenotype promote proliferation and differentiation of NPCs and increase neuronal densities in experimental stroke models.^108^, ^109^, ^110^ An appropriate inflammatory milieu is likely to provide a suitable microenvironment for neurogenesis but detailed mechanisms underlying the inflammatory responses that regulate neurogenesis remain unknown. Furthermore, microglia might also influence neurogenesis by regulating the migration of NPCs. Recently, a distinct population of microglia with special genetic profiles were shown to assist in the migration of NPCs from the SVZ and integration of NPCs into other brain regions, but these mechanisms remain to be verified in stroke models. ^111^

### 4.3 Impact on angiogenesis

The impact of microglia/macrophage activation on post‐stroke neurovascular remodeling and angiogenesis predominantly depends upon polarization phenotype.^106^ Titrating microglia/macrophage activation to an anti‐inflammatory phenotype may facilitate angiogenesis in experimental stroke.^112^, ^113^, ^114^, ^115^ A recent study transplanted IL‐4 (M2)‐polarized BV2 microglia into mouse ischemic brains and identified higher angiogenin expression.^116^ VEGF is a major participant in the regulation of normal and pathological angiogenesis. Microglia/macrophages mediate vascular sprouting via the VEGF signaling pathway by directly expressing VEGF isoforms at the later phases of stroke.^117^ Moreover, microglia can also indirectly promote the release of VEGF‐A and platelet‐derived growth factor‐BB (PDGF‐BB) from endothelial cells, thereby enhancing angiogenesis in microglia and endothelial co‐culture cell systems.^118^

### 4.4 Impact on white matter integrity

At acute phases of stroke, overexpression of pro‐inflammatory factors such as iNOS and TNF‐α by activated microglia/macrophages may play a pivotal role in damaging oligodendroglial progenitor cells (OPCs) and oligodendrocytes (OLs), resulting in white matter impairment.^119^, ^120^, ^121^ However, shifting microglia/macrophages toward M2‐phenotypes may promote white matter integrity and oligodendrogenesis in the sub‐acute or chronic phases after stroke.^58^, ^122^, ^123^, ^124^, ^125^ For example, deletion of Na^+^/H^+^ exchanger, Nhe1, in microglia/macrophages successfully promotes the expression of anti‐inflammatory cytokines, including Ym1, TGF‐β, and IL‐10, which subsequently stimulates white matter remyelination at 14 days after stroke.^126^ In addition, the phagocytic capabilities of microglia/macrophages enable engulfment of myelin debris and provide neuroprotection in stroke.^127^ In an experimental ICH model, white matter fibers within the hematoma may be used by microglia/macrophages as a scaffold to infiltrate into the hematoma and assist in its clearance.^128^

### 4.5 Impact on neuroplasticity

Neuroplasticity after stroke includes not only restoration of neural networks and circuitry, but also rewiring of functional connections. In recent years, there is a growing consensus that microglia/macrophages play a central role in regulating neuroplasticity, although the exact underlying mechanisms remain poorly understood. Experimental evidence suggests that microglia/macrophages can rapidly modify neuronal activity and modulate synaptic function, thereby supporting recovery from stroke injuries.^129^ After transient experimental cerebral ischemia, the duration of contact between microglia/macrophage processes and synapses is substantially increased and is frequently followed by the removal of the presynaptic boutons.^130^ Further, some synapses in ischemic areas disappear after the establishment of these prolonged microglia‐neuron interactions, indicating that these glial cells support an increase in synaptic turnover.^130^ Functionally, a heightened neuroinflammatory response in microglia/macrophages can rapidly modify neuronal activity and modulate synaptic function. Specifically, it was found that activation of microglia/macrophages by an inflammatory stimulus may drive long‐term synaptic depression (LTD) in neurons by NADPH oxidase, one of the main mediators of neurotoxicity in stroke.^131^ Studies examining the differential impact of microglia/macrophage phenotypic polarization on synaptic plasticity after stroke are needed.

## 5 THERAPEUTIC OPPORTUNITIES AND CHALLENGES IN TARGETING MICROGLIA/MACROPHAGES IN STROKE

### 5.1 Cell‐based therapy: Microglial transplantation and alloreplacement

Microglia transplantation or alloreplacement (by non‐self cells) has been proposed to attenuate brain injury and improve neurological outcomes after stroke. In a rat MCAO model, exogenous microglia injected into the cerebral ventricles migrate to the lesion site, reducing the associated neurodegeneration and behavioral deficits.^132^, ^133^ In chronic cerebral ischemia, transplantation of HMO6 cells (a human microglia cell line) inhibited ischemia‐induced white matter damage by reducing MMP‐2 in microglia.^134^ Although these studies transplanted untreated microglia, others advocate use of polarized (typically M2) microglia in preclinical models. Transplantation of IL‐4‐pretreated BV2 microglia into recipient mice 45 minutes after MCAO ameliorated ischemia‐induced brain damage and promoted angiogenesis.^116^ Thus, microglial transplantation has emerged as a novel therapeutic strategy to ameliorate stroke injury and improve neurological function. However, some studies have shown contradictory results. In a permanent cerebral ischemia model, rats were injected with microglia through the tail vein at 24 hours after stroke injury. These cells, however, failed to protect the brain and improve neurological function.^135^ These discrepancies might be because of differences in animal models, transplantation strategies, intervention delivery routes and regimens, and other features of the experimental paradigms. In addition to direct transplantation of microglia, some studies have explored cell‐based therapies by transferring monocytes/macrophages,^136^ bone marrow stromal cells,^137^ umbilical cord cells,^138^, ^139^ and mesenchymal stem cells^140^ into recipient animals after stroke; the transplanted cells modulated inflammatory responses and exerted neuroprotection.

One significant limitation of the traditional transplantation method is the low replacement efficiency, which may be another reason for inconsistent therapeutic outcomes of microglia transplantation or alloreplacement. In 2020, Xu et al. reported three highly efficient strategies for microglia replacement globally in the CNS or in specific brain regions.^141^ Compared to traditional methods, they created niches in the mouse brain that were microglia‐free, prior to non‐self cell transplantation. Specifically, PLX5622‐formulated chow and whole‐body irradiation were employed to deplete CNS‐resident microglia before transplantation. These microglia‐free niches aided survival and engraftment of transplanted cells. The transplantation strategies also open up a window for treatment of microglia‐associated CNS disorders, aside from stroke.^141^ Although microglia alloreplacement showed therapeutic promise, more studies are needed to understand the exact role, mechanism, and integration of grafted microglia and their impact on outcomes after stroke.

### 5.2 Harnessing the protective molecules synthesized and released by microglia/macrophages

Protective microglia/macrophages produce beneficial molecules to attenuate brain damage and promote recovery after stroke. For example, TGF‐α derived by M2‐microglia enhances proliferation and differentiation of NPCs in the SVZ of mice after cerebral ischemia.^107^ Exosomes derived from M2‐microglia/macrophages carry molecules such as miR‐124 that can protect mice from post‐stroke cognitive impairment and promote functional recovery.^142^, ^143^, ^144^, ^145^ The matricellular glycoprotein osteopontin (OPN) produced by infiltrating macrophages re‐establishes the integrity of the BBB after ischemic stroke.^146^ Similarly, inhibitors that block the detrimental factors produced by microglia/macrophages can also achieve neuroprotection after stroke. For example, adiposomes derived from microglia have pro‐inflammatory and/or pro‐death effects in ischemic brains, and inhibiting adiposome formation with chemicals such as NS‐398 reduces neuroinflammation, brain infarct volume, and motor deficits.^147^ M1‐phenotype microglia secrete TNF‐α, which may disintegrate the BBB and induce endothelial necroptosis after ischemic stroke. However, infliximab, a drug that inhibits TNFα, reduces these pathologies and improves neurological scores.^100^ Despite these studies demonstrating the therapeutic potential of modulating microglia‐derived soluble factors, many challenges remain to translate these findings into clinical application.

### 5.3 Gene correction or manipulation of intracellular phenotypic switches in microglia/macrophages

Recent studies have identified several intracellular molecular switches that control phenotypic changes in microglia/macrophages. Precise gene correction or manipulation of these switches may lead to novel therapies that boost repair functions of microglia/macrophages after stroke. miRNAs are important intracellular molecular switches that regulate microglial phenotypic change.^148^ As described above, miRNA‐124 is one such potential miRNA for stroke therapy. Injection of miRNA‐124 to mice with stroke injury shifts the polarization of microglia from pro‐inflammatory to the anti‐inflammatory phenotype and upregulates the expression of Arg‐1, which is associated with neuronal protection and post‐stroke recovery.^149^, ^150^ On the other hand, the well‐characterized miRNA‐155 promotes microglia M1 polarization, and siRNA‐mediated knockdown of miRNA‐155 in BV2 microglial cells mitigates pro‐inflammatory damage induced by LPS.^151^ In addition to miRNAs, strategies targeted at modulating the expression of other molecules, such as STATs and PPARγ, also have the potential to promote stroke recovery.^56^, ^152^

Precise gene correction in microglia offers important insights into neurological diseases. Triggering receptor expressed on myeloid cells 2 (TREM2) is upregulated in microglia of mice subjected to MCAO.^153^ Knockdown of microglial TREM2 intensifies the pro‐inflammatory response and exacerbates brain injury, due perhaps to the phagocytic function of TREM2, which helps clear apoptotic cell debris.^154^ For precise gene correction, adeno‐associated viruses (AAVs) and retroviruses are most commonly used as vehicles for gene delivery. Intrahippocampal injection of AAV particles containing RNAi for silencing cyclin‐dependent kinase 5 (CDK5) prevents microglial hyperreactivities, hippocampal degeneration, and cognitive dysfunction after cerebral ischemia.^155^ However, as AAV does not specifically target microglia per se, translatable strategies employing recombinant AAVs with microglia cell‐specific, promoter‐driven expression are urgently needed.

### 5.4 Repopulation/rejuvenation of microglia/macrophages

Microglia/macrophage repopulation or rejuvenation by genetic or pharmacological tools has potential in modulating neurological functions in different brain disorders.^156^, ^157^ In 24‐month‐old mice, depletion and repopulation of microglia with the colony‐stimulating factor 1 (CSF1) receptor inhibitor PLX5622 improves spatial memories and reverses age‐related changes in gene expression in neurons.^158^ Repopulation of microglia in organotypic hippocampal slice culture after removal of PLX3397, another widely used CSF1 receptor inhibitor, provides an anti‐inflammatory, trophic environment and returns pro‐inflammatory cytokines to normal levels.^159^ The source of the repopulated microglia remains an obvious and important question to answer. Some studies support the view that peripheral myeloid cells may be engrafted to occupy the CNS microglial niches after microglia depletion.^160^, ^161^ Others believe that microglial repopulation relies on self‐renewal of CNS‐resident microglia.^162^, ^163^ In either case, there is general agreement that freshly repopulated microglia tend to exhibit a neuroprotective and pro‐regenerative phenotype, which facilitates brain repair and alleviates neurological deficits after aging or brain injury.

In a model of ~80% hippocampal neuronal loss, microglial elimination followed by repopulation promoted recovery of cognitive function in mice by regulating synaptic plasticity.^164^ In traumatic brain injury (TBI), although microglial depletion does not alter cognitive performance, repopulating microglia attenuate cognitive deficits and promote neurogenesis via IL‐6 trans‐signaling.^165^ CSF1R inhibition effectively and sustainably depletes microglia in two experimental models of ICH (induced by injection of collagenase or autologous blood) and attenuates ICH‐induced neurological deficits and edema by promoting BBB integrity.^166^ In transient focal cerebral ischemia, depletion of microglia by administration of PLX3397 for 21 days exacerbates neurological deficits and brain infarction at 24 hours after MCAO.^167^, ^168^ However, microglial repopulation occurs within 2 weeks after withdrawing PLX3997 from the diet, and repopulation reverses the brain infarction induced by microglial depletion.^168^ Although microglia repopulation after depletion shows therapeutic potential against stroke, there are still limitations of the approach. For example, pharmacological and systemic depletion of microglia may have adverse effects on peripheral immune cells that also express the targeted receptor. Although CSF1R inhibitors have been examined in clinical settings (*eg*, NCT01329991, NCT01282684) for their safety and tolerability, further studies are needed to better understand the underlying mechanisms of microglia depletion and repopulation and to improve the specificity of this approach for clinical application.

### 5.5 Challenges in the therapeutic targeting of microglia in stroke injury

Although extensive preclinical studies support the therapeutic potential of targeting microglia for stroke, several hurdles remain before this approach can be translated into effective clinical use. First, microglia switch their phenotypes dynamically in a spatiotemporal pattern after brain injuries, including stroke. Therefore, therapeutic targeting of microglia phenotype must be precise in terms of ischemic phases, brain regions, and timing. Second, the molecules and signaling pathways that underlie microglial phenotypic regulation may have other functions. For example, miRNAs not only participate in microglia‐induced responses, but also multiple other neuropathological responses within other cell types. Manipulation of these factors may generate unwanted bystander effects. Although genetic tools have improved our mechanistic understanding of the contribution of microglia to stroke outcomes, these may not be feasible in patients or sufficiently safe. Third, the critical impact of aging and sex differences on microglial function are largely neglected in preclinical studies but are likely to influence clinical outcomes. Fourth, preclinical stroke models do not fully recapitulate the heterogeneity of the human disease—a major reason for failure of clinical translation of neuroprotectants. Similarly, heterogeneities exist at the cellular level, as microglial polarization phenotypes are not mutually exclusive. Finally, indiscriminate targeting of all microglia may elicit changes in nearby cells (*eg*, astroglia) to compensate for loss of innate immune function. Thus, fine‐tuning the immune response may be superior as a therapeutic approach, rather than systemic and nonspecific loss of a cell type that serves as a first‐responder to injury and disease.

## 6 SUMMARY

This review provides an overview of microglia in brain injury and repair after stroke. As the major innate immune cells of the CNS, microglia are the professional phagocytes of the brain and spinal cord and also participate in numerous developmental events, including neurogenesis and neural circuit formation. In the adult brain, microglia continue to serve as immune sentinels to monitor the microenvironment and maintain homeostasis. After stroke injury, microglia undergo rapid morphological changes, polarizing into pro‐ or anti‐inflammatory phenotypes, and steering the course of further degeneration or eventual recovery. Microglia/macrophage polarization can be regulated by several modulators, including transcription factors, microRNAs, and drug delivery. Targeting microglial phenotype through these modulators may help combat stroke injury and encourage tissue repair and functional recovery.

## CONFLICT OF INTEREST

The authors declare that they have no conflict of interest.

## Data Availability

Data sharing not applicable to this article as no datasets were generated or analyzed during the current study.
